# Preoperative Breast MRI in Surgical Decision-Making for Breast Cancer: Clinical Value Beyond Sensitivity

**DOI:** 10.3390/cancers18101561

**Published:** 2026-05-12

**Authors:** Luigi Schiavone, Iliana Bednarova, Marcella Buono, Chiara Dal Bosco, Domenico Ruggieri, Lucia Pilati, Massimo Ferrucci, Roberto Grassi, Francesca Caumo

**Affiliations:** 1 Radiology Unit, Department of Precision Medicine, University of Campania “Luigi Vanvitelli”, 80138 Naples, Italyroberto.grassi@unicampania.it (R.G.); 2 Unit of Breast Radiology, Veneto Institute of Oncology IOV-IRCCS, Via Gattamelata, 35128 Padua, Italy; 3 Department of Interventional and Emergency Radiology, San Giuseppe Moscati Hospital, 83100 Avellino, Italy; 4 Breast Surgery Unit, Veneto Institute of Oncology IOV–IRCCS, 35128 Padua, Italy; massimo.ferrucci@iov.veneto.it; 5 Department of Breast Imaging, Pederzoli Hospital, 37019 Peschiera, Italy

**Keywords:** breast cancer, preoperative imaging, magnetic resonance imaging, contrast-enhanced mammography, surgical decision-making, mastectomy, re-excision, invasive lobular carcinoma, ductal carcinoma in situ, value-based imaging

## Abstract

**Background:** Preoperative contrast-enhanced breast imaging, particularly magnetic resonance imaging (MRI) and contrast-enhanced mammography (CEM), has become increasingly integrated into the surgical management of breast cancer. Although these techniques improve detection of multifocal, multicentric, and contralateral disease, their clinical value cannot be defined by sensitivity alone. Methods: This article was conceived as a critical narrative review supported by a targeted literature search primarily conducted in PubMed/MEDLINE, with additional cross-checking in Scopus and reference lists. English-language studies addressing preoperative MRI or CEM in relation to surgical planning, re-excision, mastectomy, additional disease detection, and oncologic outcomes were prioritized. Overall, 38 clinically relevant studies were included in the qualitative narrative synthesis. **Results:** A decision-oriented reading of the literature suggests that preoperative contrast-enhanced imaging does not produce a uniform clinical effect, but rather reshapes management through recurring patterns. Three main patterns can be identified: (1) refinement of surgical extent through improved disease mapping, most clearly in invasive lobular carcinoma, where MRI may reduce repeat surgery without consistently increasing mastectomy rates; (2) pre-emptive escalation aimed at avoiding reoperation, particularly in ductal carcinoma in situ, where lower re-excision rates are often accompanied by higher initial mastectomy rates; and (3) decision amplification driven by the detection of additional or indeterminate disease, frequently resulting in additional biopsies and treatment escalation without consistent evidence of long-term oncologic benefit. Across large observational cohorts, routine preoperative MRI has not been consistently associated with improvements in overall survival, disease-free survival, or locoregional control in unselected populations. These patterns should be understood as interpretive categories derived from recurring trends in a heterogeneous literature, rather than as formally validated decision classifications. **Conclusions:** The value of preoperative contrast-enhanced breast imaging depends less on sensitivity itself than on how imaging findings are translated into surgical action. Selective, question-driven use appears most valuable in settings such as invasive lobular carcinoma and assessment of nipple–areola complex involvement, whereas routine use in unselected populations often appears to increase surgical intensity more clearly than it improves long-term oncologic outcomes. Contrast-enhanced imaging should therefore be regarded as a decision-shaping tool whose clinical usefulness depends on explicit interpretive and multidisciplinary thresholds.

## Simple Summary

Preoperative contrast-enhanced breast imaging, especially magnetic resonance imaging and contrast-enhanced mammography, can reveal additional cancer foci that are not always visible on conventional imaging. However, finding more disease does not automatically mean that patients receive better care. This review examines how these imaging techniques influence surgical decisions in breast cancer, including wider excision, reoperation, mastectomy, or, in selected cases, less extensive surgery. The literature suggests that the value of these examinations depends on the clinical setting. They appear most useful when they answer a specific surgical question, such as defining disease extent in invasive lobular carcinoma or assessing whether nipple-sparing surgery is feasible. In contrast, routine use in unselected patients may increase biopsies and surgical intensity without clear improvement in long-term outcomes. A more selective, multidisciplinary, and decision-focused use of contrast-enhanced imaging may therefore improve the proportionality of care.

## 1. Introduction

Preoperative imaging in breast cancer does not simply inform surgical planning; it helps shape it. By influencing how the extent of disease is perceived, imaging contributes to whether treatment is framed as conservative or extensive, localized or multifocal, proportionate or precautionary. In this sense, the increasing use of contrast-enhanced breast imaging represents more than a technical development. It reflects a broader shift in how surgical adequacy and oncologic risk are interpreted before treatment.

Breast magnetic resonance imaging (MRI) offers the highest sensitivity among currently available imaging modalities for detecting multifocal, multicentric, and contralateral breast cancer. Across prospective studies, retrospective analyses, and population-based cohorts, MRI has repeatedly been shown to reduce radiologic underestimation of disease extent and to identify additional lesions not detected by mammography or ultrasound [1,2,3,4]. These strengths have progressively expanded its preoperative use beyond a narrow set of indications, often driven by the concern that incomplete disease mapping may lead to inadequate surgery or additional procedures.

At the same time, greater sensitivity does not automatically translate into better care. A recurrent theme in the literature is that preoperative MRI is associated with substantial changes in surgical management, including higher rates of mastectomy among patients otherwise considered eligible for breast-conserving surgery, while its effects on re-excision, local control, and survival remain inconsistent [5,6,7,8,9]. Recent prospective and randomized evidence indicates that preoperative MRI may change the overall treatment plan in a substantial proportion of selected patients and the breast surgery plan in approximately one-third, while also increasing planned mastectomy rates in some settings [1,6].

Long-term follow-up studies have not consistently shown improved overall survival, disease-free survival, or locoregional outcomes with routine preoperative MRI, raising an important question: Does detecting more disease necessarily improve the quality of surgical decision-making [10,11,12]?

This question is especially relevant in an era of increasingly individualized breast cancer treatment. Under contemporary multidisciplinary management, the clinical significance of additional radiologic disease is not always self-evident. Some findings are clearly relevant because they alter the feasibility of breast conservation or identify otherwise occult multicentric disease. Others, however, may increase diagnostic and therapeutic intensity without clearly changing patient-relevant outcomes. The challenge, therefore, is not detection alone, but the interpretation of detection within a proportional decision framework.

The impact of contrast-enhanced imaging is also highly context-dependent. In invasive lobular carcinoma, where conventional imaging often underestimates disease extent, MRI appears to provide the most consistent decision-level value by improving extent assessment and, in selected settings, reducing repeat surgery without systematically increasing mastectomy rates [13,14,15,16,17]. In ductal carcinoma in situ, by contrast, MRI improves delineation of disease extent but often shifts initial treatment toward more extensive surgery, with reductions in re-excision frequently offset by higher first-line mastectomy rates and without consistent long-term oncologic benefit [18,19,20,21,22,23].

Functional imaging may also be valuable when applied to focused surgical questions. MRI has shown a high negative predictive value for nipple–areola complex involvement and may support nipple-sparing mastectomy in selected patients, particularly when enhancement is absent or resolves after neoadjuvant therapy [24,25,26]. In these settings, imaging can facilitate surgical de-escalation rather than escalation.

Contrast-enhanced mammography (CEM) has emerged as a more accessible alternative to MRI, with high sensitivity for index lesion assessment and a potentially different balance between additional findings and downstream intervention. Available comparative studies suggest that although MRI may detect more additional malignant foci, CEM may influence surgical planning with fewer indeterminate findings and less additional workup [27,28,29,30].

Most published reviews on preoperative contrast-enhanced breast imaging have focused primarily on diagnostic performance and lesion detection. However, the central clinical issue is no longer whether these techniques detect more disease, but how that additional information changes management and whether those changes are proportionate, beneficial, and clinically meaningful. The aim of this review is therefore to examine preoperative contrast-enhanced breast imaging through a decision-oriented lens, with particular emphasis on surgical planning, treatment escalation or refinement, long-term oncologic implications, and value-based clinical use.

## 2. Scope and Approach of This Narrative Review

This article was conceived as a critical narrative review aimed at examining how preoperative contrast-enhanced breast imaging influences surgical decision-making in breast cancer. The objective was not to provide an exhaustive or quantitative synthesis of all published studies, but to offer a clinically oriented interpretation of the literature centered on management consequences rather than diagnostic performance alone.

The interpretive framework adopted in this review is decision-oriented. Rather than organizing the literature primarily by imaging modality or technical performance, the analysis is structured around recurring ways in which increased imaging sensitivity reshapes surgical choices. This approach was chosen to clarify when contrast-enhanced imaging appears to refine management in a proportionate way and when it may instead contribute to treatment intensification without clearly demonstrated patient-relevant benefit.

The proposed framework should be understood as an interpretive structure derived from recurring trends across a heterogeneous literature, rather than as a formally validated classification system.

### Search Strategy and Study Selection

To support this narrative appraisal, a targeted literature search was primarily conducted in PubMed/MEDLINE, with additional cross-checking in Scopus and by reviewing the reference lists of relevant articles. The search focused on English-language studies addressing preoperative breast magnetic resonance imaging (MRI) or contrast-enhanced mammography (CEM) in relation to surgical planning, treatment escalation or de-escalation, re-excision, mastectomy, additional disease detection, and oncologic outcomes. Search terms combined concepts related to breast cancer, preoperative MRI, contrast-enhanced mammography, surgical management, re-excision, mastectomy, multifocal or contralateral disease, and survival outcomes.

Approximately 1000 records were initially identified and screened for relevance. Studies were then selected on the basis of clinical relevance and direct contribution to the review question, with particular attention to meta-analyses, population-based cohorts, matched observational studies, randomized data where available, and selected institutional series with clear management implications. Studies focused primarily on technical imaging performance without clinical management relevance, case reports, and papers not directly addressing the preoperative setting were not prioritized for inclusion. Overall, 38 studies were considered most relevant and were included in the qualitative narrative synthesis.

Because the aim of this article is interpretive rather than exhaustive, study selection was guided by clinical relevance and decision-making impact rather than by formal systematic review methodology. No predefined protocol, duplicate screening process, formal risk-of-bias assessment, or quantitative synthesis was undertaken.

**Figure 1:**
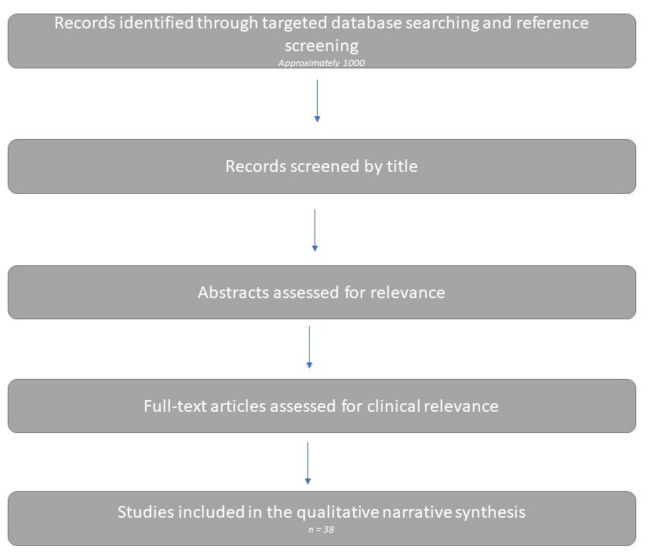
Simplified overview of literature identification and study selection for the narrative review.

A simplified overview of the literature identification and study selection process is provided in Figure 1.

## 3. Why Increased Sensitivity Changes Surgery

The surgical impact of contrast-enhanced breast imaging arises from a simple mechanism: it changes the apparent extent of disease before treatment decisions are finalized. In breast cancer, surgical planning depends heavily on the perceived distribution of disease within the breast, the suspicion of occult multifocality or multicentricity, the relationship between tumor extent and cosmetic feasibility, and the possibility of contralateral involvement. Any imaging modality that alters this map also alters the decision environment in which surgery is chosen.

**Figure 2:**
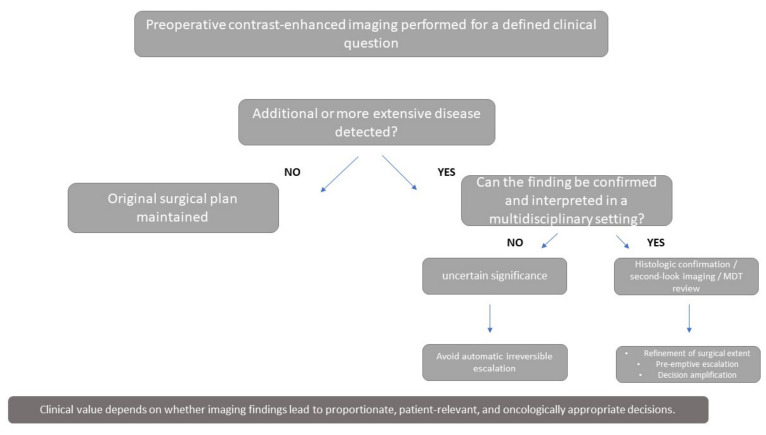
Decision pathway illustrating how preoperative contrast-enhanced breast imaging may influence surgical management in breast cancer. Preoperative MRI or CEM may be used to address a defined clinical question, such as extent assessment, feasibility of breast conservation, nipple–areola complex preservation, or clarification of discordant conventional imaging. When additional or indeterminate findings are detected, their management should depend on the availability of confirmatory pathways, including second-look imaging, biopsy, and multidisciplinary review. Imaging may support refinement of surgical extent, pre-emptive escalation to avoid reoperation, or decision amplification through additional lesions of uncertain significance. The clinical value of imaging depends not only on detection, but on whether acting on imaging findings improves proportionate, patient-relevant, and oncologically appropriate decision-making. The three pathways shown should be understood as interpretive categories rather than formally validated decision classifications.

Because preoperative decisions are often made under conditions of incomplete certainty, greater sensitivity may either refine planning or increase decisional complexity, depending on the clinical setting and the threshold at which additional findings trigger a change in treatment. A useful way to interpret the literature is therefore to move beyond the binary question of whether preoperative contrast-enhanced imaging is beneficial or not, and instead ask how it changes decisions. From this perspective, three recurrent decision patterns can be identified across published studies: refinement of surgical extent through improved disease mapping, pre-emptive escalation to avoid reoperation, and decision amplification driven by the detection of additional or indeterminate disease. This conceptual framework is illustrated in Figure 2.

## 4. Three Decision Patterns Associated with Preoperative Contrast-Enhanced Imaging

A decision-oriented reading of the literature suggests that the clinical impact of preoperative contrast-enhanced breast imaging is not uniform. Rather than producing a single type of benefit or harm, MRI and CEM appear to reshape management through recurrent patterns of decision-making. These patterns are influenced by the interaction between imaging sensitivity, tumor biology, baseline surgical intent, and the clinical thresholds used to translate new findings into action.

Across published studies, three broad decision patterns can be distinguished. The first is refinement of surgical extent through improved disease mapping, in which additional imaging information helps align surgery more closely with actual pathologic extent. The second is pre-emptive escalation to avoid reoperation, in which the desire to reduce margin positivity or repeat surgery leads to a more extensive initial procedure. The third is decision amplification through the detection of additional or indeterminate disease, in which greater sensitivity increases the number of findings requiring interpretation and often shifts care toward additional biopsies or more extensive treatment.

These patterns are not mutually exclusive, and more than one may coexist within the same clinical context. Nevertheless, they offer a practical framework for distinguishing situations in which increased sensitivity appears to improve decision quality from those in which it more often intensifies treatment without a clearly demonstrated patient-level benefit. These patterns are summarized in Table 1.

Representative references supporting the clinical interpretations summarized in this table include [1,6,10,13,14,15,16,20,21,23,28,30].

The proposed decision patterns should be understood as an interpretive framework derived from recurring trends across heterogeneous studies, rather than as formally validated categories.

### 4.1. Refinement Through Improved Disease Mapping

The clearest example of imaging-driven refinement is found in settings where conventional imaging is known to underestimate the true extent of disease. In such cases, contrast-enhanced imaging may correct a structurally incomplete preoperative assessment and allow surgery to be planned more appropriately from the outset.

This pattern is most evident in invasive lobular carcinoma (ILC). Because of its infiltrative growth pattern and often subtle mammographic or sonographic appearance, ILC is frequently underestimated on conventional imaging. Across retrospective series, matched analyses, and population-based data, preoperative MRI has repeatedly been shown to identify additional ipsilateral disease and to alter the planned surgical approach in a substantial proportion of patients with lobular cancer, with management changes reported in roughly one-fifth to one-third of cases across institutional and matched series [13,14,15,16,17]. Importantly, in this setting, the additional information provided by MRI appears more likely to represent clinically meaningful extent rather than incidental burden. In matched and observational ILC cohorts, MRI-detected additional lesions have been reported in approximately 39–45% of patients, with malignant confirmation in 58–66% of suspicious findings, management changes in about 10–26%, and reduced repeat surgery without a consistent increase in mastectomy rates [13,14,16].

Several studies suggest that when MRI is used selectively in ILC and imaging-detected findings are incorporated into a defined surgical plan, repeat surgery may be reduced without a corresponding increase in mastectomy rates [14,15,16,17]. This is a crucial distinction. The benefit in this scenario does not arise from imaging simply detecting more lesions, but from improving the fit between the initial operation and the underlying pattern of disease. In other words, imaging acts as a corrective tool rather than an escalation trigger.

A similar form of refinement has been described in relation to nipple–areola complex (NAC) assessment. Here, the question is not whether more disease exists somewhere in the breast, but whether a specific anatomic structure can be safely preserved. MRI has shown a high negative predictive value for NAC involvement and may support nipple-sparing mastectomy when enhancement between the index lesion and the nipple is absent or resolves after neoadjuvant therapy [24,25,26]. In this context, contrast-enhanced imaging may facilitate a less extensive yet oncologically acceptable operation. This is one of the most convincing examples of value-aligned imaging: the information obtained addresses a focused surgical question and directly enables de-escalation.

The key feature of this first pattern is that increased sensitivity supports better calibration of surgical extent, rather than simply greater treatment intensity. It is therefore most likely to be clinically valuable when disease underestimation is common, the surgical question is well defined, and the pathway from imaging finding to operative decision is clear.

### 4.2. Pre-Emptive Escalation to Avoid Reoperation

A second recurrent pattern is one in which contrast-enhanced imaging reduces uncertainty by favoring a more extensive initial operation. Here, the main clinical objective is not necessarily better matching of surgery to pathologic extent, but avoidance of positive margins, re-excision, or subsequent conversion to mastectomy. This pattern is particularly evident in ductal carcinoma in situ (DCIS).

MRI improves the delineation of disease extent in DCIS and reduces radiologic underestimation compared with conventional imaging alone [18,19,20]. Several observational studies, matched analyses, and meta-analyses have reported lower re-excision rates in patients undergoing preoperative MRI, often with reductions into the approximate 8–12% range but at the cost of higher initial mastectomy rates [19,20,21,22,23]. In DCIS, the quantitative trade-off is particularly important: randomized and matched data suggest reoperation rates of approximately 10–20% with MRI versus 22–27% without MRI in selected cohorts, whereas first-line mastectomy may increase from about 11% to 20% [20,21,23].

On the surface, this appears to support a beneficial role for imaging. However, the reduction in repeat surgery is frequently accompanied by higher rates of initial mastectomy, suggesting that the apparent gain may often be achieved through earlier escalation rather than through more precise breast-conserving surgery [20,21,22,23].

This distinction matters clinically. A lower re-excision rate is not automatically synonymous with better care if it is consistently obtained at the cost of a more extensive first operation. In DCIS, MRI may improve anatomic delineation but does not reliably distinguish lesions that truly require wider surgery from those that remain suitable for conservative treatment. As a result, improved visualization of extent may translate into greater surgical certainty without necessarily improving proportionality of treatment.

The implications of this pattern extend beyond DCIS. In mixed invasive populations, reduced reoperation rates associated with preoperative MRI have also been reported alongside increased mastectomy rates [6,7,31]. In these situations, contrast-enhanced imaging may function less as a tool of refinement than as a means of lowering tolerance for residual uncertainty. The consequence is a form of pre-emptive escalation: a broader initial procedure is selected in order to minimize the possibility of a second intervention.

This pattern should not be interpreted as evidence that imaging is intrinsically harmful. In some patients, avoiding repeat surgery may be a meaningful benefit. However, the trade-off needs to be made explicit. The relevant question is not only whether MRI reduces re-excision, but how that reduction is achieved and whether the surgical exchange is acceptable in light of oncologic, functional, and patient-centered outcomes.

### 4.3. Decision Amplification Through Additional or Indeterminate Disease

A third pattern emerges when increased sensitivity expands the number of actionable findings without providing equally clear guidance about their clinical significance. In this scenario, contrast-enhanced imaging does not simply clarify the extent of the known cancer; it generates additional lesions, indeterminate enhancement, or contralateral abnormalities that require further workup and may alter treatment trajectories.

This pattern is most familiar in the setting of additional ipsilateral or contralateral findings detected on preoperative MRI. Across heterogeneous studies, MRI identifies previously occult lesions in a substantial minority of patients, often in the approximate 15–45% range across heterogeneous series, and a meaningful proportion of biopsied findings prove malignant [1,2,3,32,33,34]. At a technical level, this supports the validity of the modality. Yet from a decision standpoint, the issue is more complex. Additional findings frequently initiate further imaging, second-look ultrasound, biopsy, multidisciplinary reassessment, and changes in surgical planning, including conversion to mastectomy or bilateral surgery [1,2,3,9,33].

This process can be clinically justified when additional disease is confirmed and clearly alters the feasibility or oncologic adequacy of the original surgical plan. The difficulty arises when the pathway from detection to action is less stable. Non-mass enhancement (NME) is a prominent example. Compared with more discrete mass-like findings, NME often has more variable positive predictive value, is less consistently matched on second-look ultrasound, and may be more likely to trigger prolonged diagnostic workup or precautionary treatment escalation [35]. In such cases, imaging increases decisional burden as much as it increases certainty.

For this reason, the effect of preoperative contrast-enhanced imaging in this third pattern may be understood as decision amplification. Imaging strengthens the visibility of potential disease, but it also broadens the set of findings that can influence care. The more lesions that enter the decisional field, the greater the chance that management will be intensified, even when the long-term patient benefit of that intensification remains uncertain. This helps explain why management changes after MRI are often pathologically understandable yet still fail to translate into consistent improvements in survival or locoregional outcomes [10,11,12].

This pattern does not argue against the importance of detecting occult disease. Rather, it highlights the need for a more disciplined interpretive framework. When additional findings are discovered, the key clinical question is not only whether they are real, but whether and under what conditions they should change irreversible surgical decisions.

## 5. Clinical Contexts in Which Imaging Adds the Most Value

The decision-level value of preoperative contrast-enhanced imaging is not evenly distributed across breast cancer care. Its contribution depends on the likelihood that conventional imaging underestimates disease, the specificity of the surgical question being asked, and the extent to which additional findings can be incorporated into management without automatically driving overtreatment. A context-based approach is therefore more clinically useful than judging MRI or CEM as universally beneficial or universally excessive. The clinical contexts in which preoperative contrast-enhanced imaging is more or less likely to add decision-level value are summarized in Table 2.

Representative references supporting the contexts summarized in this table include [13,14,15,16] for invasive lobular carcinoma, [20,21,23] for ductal carcinoma in situ, [24,25,26] for nipple–areola complex assessment, and [28,29,30] for contrast-enhanced mammography.

### 5.1. Invasive Lobular Carcinoma

Among all indications, invasive lobular carcinoma is the setting in which preoperative MRI appears to have the strongest and most consistent rationale. Conventional imaging frequently underestimates the extent of lobular tumors because of their growth pattern, lower conspicuity, and tendency toward multifocality or multicentricity. In this context, MRI is more likely to reveal disease that is both anatomically relevant and surgically consequential [13,14,15,16,17].

Importantly, the value of MRI in ILC is not limited to detecting more disease. Several studies suggest that MRI may reduce repeat surgery without a parallel increase in mastectomy, particularly when used selectively and when additional findings are interpreted within a defined operative strategy [14,15,16,17]. Population-based data also support a histology-dependent effect, with MRI appearing to increase mastectomy rates in ductal cancers while reducing them in lobular cancers [13]. For this reason, ILC can be regarded as the most convincing example of decision-relevant use of preoperative contrast-enhanced imaging in current practice.

### 5.2. Ductal Carcinoma In Situ

The role of preoperative MRI in ductal carcinoma in situ is more problematic. MRI improves the delineation of disease extent and can reduce radiologic underestimation [18,19,20]. It may also lower re-excision rates [19,20,21,22,23]. However, these apparent advantages are frequently accompanied by increased first-line mastectomy rates and without a consistent signal for improved long-term oncologic outcomes [6,21,22,23].

In DCIS, better visualization of extent does not necessarily imply better biological discrimination. A broader area of enhancement may indicate a more extensive intraductal process, but it does not by itself establish that a more radical operation is needed to improve meaningful outcomes. Accordingly, MRI in DCIS should be interpreted with greater caution than in ILC. It may be useful in selected cases, particularly when conventional imaging is discordant or extent is genuinely uncertain, but the literature does not support a simple equation between more complete imaging and more appropriate surgery.

### 5.3. Assessment of the Nipple–Areola Complex and Feasibility Questions

Preoperative contrast-enhanced imaging appears especially valuable when it is used to answer a focused feasibility question rather than to survey the breast more broadly for additional disease. Assessment of the nipple–areola complex is one such example. MRI can help estimate the likelihood of NAC involvement and may support eligibility for nipple-sparing mastectomy when no suspicious enhancement is present or when previously detected enhancement resolves after neoadjuvant therapy [24,25,26].

This use case illustrates a different model of value. Here, imaging narrows uncertainty around a specific operative decision rather than broadly expanding the field of disease. The consequence may be preservation rather than escalation, making NAC assessment one of the clearest high-value indications for contrast-enhanced imaging in the preoperative setting.

### 5.4. Additional Lesions and Indeterminate Enhancement

The value of preoperative contrast-enhanced imaging becomes more uncertain when the main effect of the examination is the identification of additional lesions or indeterminate enhancement patterns. Although many of these findings are real and some are malignant, their management implications are not always proportional to their detection. This is particularly relevant when findings are visible only on MRI, when second-look ultrasound is negative, or when enhancement patterns such as non-mass enhancement introduce uncertainty without a clear pathway to conservative confirmation [35].

In these situations, the threshold for changing surgery should be especially cautious, because such findings function more as context-dependent risk signals than as stand-alone determinants of management. Histologic confirmation should precede irreversible escalation whenever feasible. Without that discipline, imaging may transform a technically valid detection into a management cascade whose net patient benefit remains unclear.

### 5.5. Contrast-Enhanced Mammography as a Different Balance of Benefit and Burden

The available literature on contrast-enhanced mammography is smaller than that on MRI, but it suggests a related decision logic. CEM has shown high sensitivity for index lesion evaluation and may influence surgical management in a meaningful minority of cases [27,28,29,30]. Comparative studies indicate that MRI often identifies more additional malignant foci, particularly multifocal disease, whereas CEM may generate fewer indeterminate findings and less downstream workup [28,29,30]. Comparative evidence remains limited, but in a prospective head-to-head study MRI detected all six additional malignant lesions whereas CEM detected one of six, despite similar sensitivity for the index lesion (97% vs. 99%), underscoring a possible trade-off between broader detection and lower false-positive burden [28].

**Figure 3:**
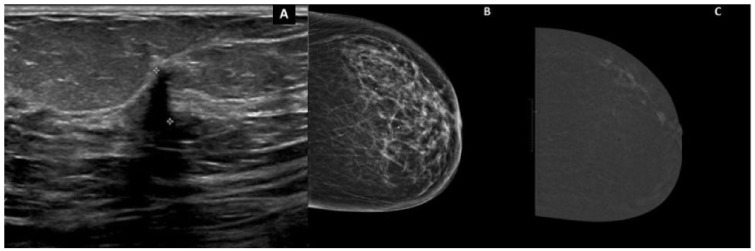
Illustrative case of invasive lobular carcinoma better depicted by contrast-enhanced mammography. The patient presented with clinical nipple thickening, and biopsy confirmed invasive lobular carcinoma. (**A**) Targeted ultrasound identified an additional corresponding lesion. (**B**) Low-energy MLO CEM image. (**C**) Corresponding recombined CEM image showing enhancement and clearer depiction of disease extent. This case illustrates a setting in which contrast-enhanced imaging may add clinically relevant information beyond conventional assessment.

This suggests that CEM may offer a different balance between information gain and decisional burden. In selected settings, it may provide sufficient functional information to support preoperative planning while limiting some of the cascade effects associated with MRI. At the same time, CEM does not resolve the underlying interpretive challenge. Its value, like that of MRI, depends on indication, context, and the discipline with which findings are translated into management. Current conclusions regarding CEM remain more provisional than those regarding MRI, given the smaller evidence base and the relative scarcity of long-term outcome data. An illustrative case is shown in Figure 3.

### 5.6. Current Guideline Context

The interpretation proposed in this review can be positioned within current breast imaging and oncology guidance, which generally supports a selective rather than routine use of preoperative breast MRI. EUSOBI recommendations have long emphasized the role of MRI as a problem-solving tool when conventional imaging is inconclusive, and as a particularly useful modality in preoperative staging when disease extent may be underestimated, especially in dense breasts, invasive lobular carcinoma, or suspected multifocal or multicentric disease. At the same time, these recommendations also highlight that additional MRI-detected foci are not uniformly malignant and that major changes in surgical management should, whenever feasible, be supported by histologic confirmation.

More recent ESMO guidance is aligned with this selective approach, recommending breast MRI in the presence of uncertainties after standard imaging and in specific clinical situations, including lobular cancers, suspected multifocality or multicentricity, hereditary high-risk settings, and the presence of breast implants. Taken together, these guideline positions reinforce the central conclusion of the present review: the value of preoperative contrast-enhanced imaging lies less in routine escalation of detection than in its use as a targeted decision-support tool in clearly defined clinical scenarios [36,37].

## 6. Long-Term Oncologic Outcomes and the Limits of Anatomical Completeness

Preoperative contrast-enhanced imaging does not determine the correct operation by itself; rather, it defines a more complex decisional field within which proportional surgical judgment must be exercised. This is particularly relevant given the persistent gap in the literature between greater anatomic detection and uncertain long-term oncologic benefit.

Although MRI in particular often changes surgical management, large observational cohorts and long-term follow-up studies have not consistently shown corresponding improvements in overall survival, disease-free survival, or locoregional control in unselected patient populations [10,11,12]. Importantly, available cohort analyses have generally shown adjusted hazard ratios close to unity for recurrence-free and overall survival, supporting the view that increased preoperative detection does not automatically translate into measurable long-term oncologic benefit [10,12].

This observation does not imply that preoperative imaging lacks clinical relevance. Rather, it indicates that the relationship between detecting more disease and improving outcomes is neither linear nor automatic. Breast cancer treatment is delivered within a multimodality framework that includes surgery, radiotherapy, systemic therapy, and increasingly individualized risk stratification. Within this setting, the discovery of additional foci may alter surgical extent without necessarily changing the patient’s long-term oncologic trajectory.

This distinction matters because imaging studies are often interpreted through the lens of technical success. A modality that reveals occult multifocality, detects contralateral malignancy, or more accurately defines tumor extent is performing well diagnostically. However, diagnostic success does not by itself establish therapeutic advantage. Greater surgical extent may improve anatomical clearance without necessarily translating into measurable gains in long-term oncologic outcomes, particularly within contemporary multimodality treatment pathways in which imaging contributes probabilistic rather than deterministic decision signals.

The literature on preoperative MRI illustrates this tension clearly. Across population-based studies and institutional cohorts, MRI is frequently associated with modifications in surgical planning, including increased use of mastectomy or bilateral surgery, yet these changes have not translated into a reproducible survival advantage [10,11,12]. This does not necessarily mean that all such changes are unwarranted. Some may be pathologically justified, technically appropriate, or aligned with patient preferences. It does mean, however, that greater anatomical completeness should not be assumed to confer oncologic superiority.

Routine preoperative MRI nevertheless remains appealing in clinical practice for understandable reasons. Surgeons and radiologists are often confronted with the risk of disease underestimation on conventional imaging, the burden of re-excision, and the desire to plan the most definitive operation from the outset. In this context, a more comprehensive depiction of disease extent may appear intuitively preferable, particularly in younger patients, in dense breasts, or when conventional imaging is discordant. Preoperative MRI may also increase decisional confidence within the multidisciplinary team and may be perceived as a way to reduce unexpected findings at surgery. These considerations help explain why routine MRI use persists even in the absence of consistent evidence of long-term oncologic benefit.

What emerges from the literature is therefore a more nuanced conclusion. Preoperative contrast-enhanced imaging can meaningfully alter surgery, but the fact that surgery changes does not in itself establish that care has improved. The unresolved question is whether increased detection produces proportionate benefit at the patient level. That benefit may include fewer reoperations, more accurate selection for breast-conserving or nipple-sparing approaches, reduced unexpected disease burden at surgery, or greater decisional confidence, particularly when improved preoperative extent estimation helps avoid both under-treatment and unnecessary escalation [38]. Yet these benefits remain uneven across clinical contexts and are not adequately captured by anatomical gain alone.

For this reason, the main limitation of routine preoperative contrast-enhanced imaging in unselected populations is not simply that it detects more disease, but that it lacks sufficiently validated thresholds linking new radiologic information to meaningful differences in patient outcome. Until those thresholds are better defined, the literature supports a cautious distinction between seeing more disease and improving care.

## 7. Implications for Multidisciplinary Practice

If the value of preoperative contrast-enhanced imaging depends on how findings are translated into action, then the central challenge is not technological but multidisciplinary. MRI and CEM should not be understood as stand-alone arbiters of surgical extent, but as imaging tools that inform proportional surgical judgment within a broader multidisciplinary context. This view is consistent with recent review literature in DCIS imaging, which frames breast imaging as a probabilistic risk-filtering tool rather than a stand-alone determinant of management [39]. Their contribution becomes clinically meaningful only when imaging findings are interpreted within a framework that includes histology, baseline surgical intent, feasibility of tissue confirmation, patient priorities, and the expected impact of escalation or de-escalation on meaningful outcomes.

For radiologists, this means that preoperative reporting should move beyond lesion detection alone and become more explicitly decision-oriented. The relevant question is not only whether an additional focus enhances, but whether that enhancement is likely to alter surgical management in a justified and proportionate way. Reports that distinguish between findings likely to modify surgical extent, findings that require tissue confirmation before management change, and findings of uncertain surgical significance may help reduce automatic escalation. In particular, MRI-only lesions and non-mass enhancement should be framed with caution when no clear confirmatory pathway exists.

For breast surgeons, a more extensive radiologic map does not automatically mandate a more extensive operation. The critical step is to determine whether additional disease meaningfully changes the feasibility of breast conservation, the appropriateness of nipple preservation, or the expected oncologic adequacy of the original plan. In selected settings, such as invasive lobular carcinoma or focused nipple–areola complex assessment, preoperative MRI may clearly improve planning. In other settings, especially when the main consequence of imaging is the discovery of uncertain additional disease, the operative threshold for escalation should remain deliberate rather than reflexive.

For the multidisciplinary team, the literature strongly suggests that the clinical impact of contrast-enhanced imaging is shaped by local decision culture as much as by imaging performance itself [40]. Variation in preoperative MRI use across surgeons and institutions indicates that interpretation, risk tolerance, and practice norms play a major role in determining whether imaging leads to refinement or overtreatment. Institutions may therefore benefit from explicit pathways addressing when preoperative MRI or CEM should be obtained, which findings require biopsy before surgery is changed, and how uncertain enhancement should be discussed in tumor boards.

The issue also extends to patient counseling. Additional imaging findings can influence how patients perceive disease burden and may increase anxiety, decisional conflict, or preference for more extensive surgery even when long-term oncologic benefit is uncertain. Patients should therefore be informed that contrast-enhanced imaging may improve disease mapping in some contexts, but may also generate findings whose clinical significance is uncertain and whose detection does not always improve long-term outcomes.

Additional imaging findings may also influence patient-centered outcomes in ways that are not fully captured by conventional surgical or oncologic endpoints. Further biopsies, indeterminate lesions, and unexpected changes in surgical planning may increase anxiety, decisional conflict, treatment burden, and delay before definitive surgery. Conversely, in selected settings, improved preoperative extent assessment may reduce uncertainty, avoid reoperation, and support greater confidence in the final surgical plan. For this reason, the value of preoperative contrast-enhanced imaging should also be considered in relation to patient experience, including treatment burden, body image implications, and satisfaction with the final surgical decision, and not solely in terms of recurrence or survival metrics.

A more value-based use of preoperative contrast-enhanced imaging would therefore include several practical principles: imaging should ideally be ordered to answer a specific surgical question; irreversible escalation should, whenever feasible, be preceded by histologic confirmation of additional disease; and multidisciplinary discussion should focus not only on whether a lesion is present, but on whether acting on it is likely to improve meaningful outcomes. The practical boundaries of what preoperative contrast-enhanced imaging can and cannot reliably do in surgical decision-making are summarized in Table 3.

Representative references supporting the statements summarized in this table include [10,12,13,14,15,16,20,21,23,24,25,26,27,28,29,30,35].

The main pitfalls that may lead to disproportionate escalation are summarized in Box 1.

Box 1 **Key Interpretive Pitfalls in Preoperative Contrast-Enhanced Breast Imaging.**

○ Equating greater imaging extent with automatic need for more extensive surgery.
○ Interpreting MRI-only or non-mass enhancement findings as stand-alone triggers for escalation in the absence of confirmatory pathways.
○ Assuming that greater anatomical completeness necessarily translates into oncologic superiority.
○ Using imaging findings as deterministic classifiers rather than context-dependent decision signals.
○ Interpreting additional lesions outside their histologic, surgical, and multidisciplinary context.

To complement the conceptual summaries presented in Table 1, Table 2 and Table 3, the key studies underpinning the present decision-oriented interpretation are summarized in Table 4.

## 8. Limitations of This Narrative Review

This review has limitations that should be acknowledged. First, it was designed as a critical narrative review and not as a systematic review or meta-analysis. Accordingly, it does not provide exhaustive study capture, formal duplicate screening, risk-of-bias assessment, or pooled quantitative estimates. The interpretive framework proposed here is intended to clarify decision-level patterns in the literature rather than to generate summary effect sizes.

Second, the literature on preoperative contrast-enhanced breast imaging is highly heterogeneous. Studies differ in patient selection, tumor subtype, imaging indication, treatment era, surgical practice, endpoint definition, and length of follow-up. These differences limit direct comparability across studies and make broad generalization difficult, particularly when outcomes such as re-excision, mastectomy, or survival are interpreted across distinct clinical settings.

Third, much of the evidence base remains observational. Associations between preoperative MRI and surgical or oncologic outcomes are subject to confounding by indication, selection bias, and institutional practice effects. Patients undergoing preoperative MRI may differ systematically from those managed without MRI in ways that are not fully captured by available analyses. For this reason, apparent benefits or harms should be interpreted cautiously, especially in unselected populations.

Fourth, the conceptual distinction between refinement, pre-emptive escalation, and decision amplification is an interpretive model derived from the published literature rather than a formally validated classification system. Although this framework may help organize clinically meaningful patterns, overlap between categories is expected, and individual studies may fit more than one decisional pathway.

Finally, patient-centered outcomes remain insufficiently represented in much of the published literature. While survival, locoregional recurrence, re-excision, and mastectomy are commonly reported, outcomes such as decisional regret, anxiety related to additional findings, body image, treatment delay, and perceived quality of care are less consistently addressed. As a result, the true clinical value of preoperative contrast-enhanced imaging may be incompletely captured by currently available endpoints.

## 9. Future Directions

The next stage in evaluating preoperative contrast-enhanced breast imaging should move beyond the question of whether these techniques detect more disease. That point is already well established. The more important challenge is to determine when additional detection should alter treatment and when it should not. Future research should therefore focus less on sensitivity alone and more on the development of explicit thresholds for action.

A first priority is the creation of decision-oriented clinical frameworks that link specific imaging findings to proportionate surgical responses. This is particularly relevant for MRI-only lesions, non-mass enhancement, contralateral findings, and disease extent in ductal carcinoma in situ. Studies that examine how imaging findings are discussed in multidisciplinary settings, how often they lead to biopsy-confirmed disease, and how often they alter management in ways that improve meaningful outcomes would be especially valuable.

A second priority is the incorporation of patient-centered endpoints into future imaging research. The value of preoperative MRI or CEM cannot be judged solely by survival, recurrence, or even reoperation rates. Outcomes such as treatment burden, number of procedures, anxiety generated by additional findings, decisional conflict, body image, and satisfaction with surgical choice may provide a more complete picture of benefit and harm.

A third area for development is standardization of reporting and multidisciplinary governance. Radiology reports in the preoperative setting could become more clinically useful if they more clearly distinguish findings that are highly likely to change surgical feasibility from those that remain uncertain without tissue confirmation. Similarly, local pathways may help reduce unnecessary escalation by specifying which imaging findings should prompt additional biopsy, repeat imaging, or tumor board review before surgery is changed.

Future work should also better define the comparative role of contrast-enhanced mammography. Although current evidence suggests that CEM may offer a different balance between information gain and downstream burden, longer-term and context-specific data remain limited. Comparative studies focused on management consequences, patient experience, and resource use may help clarify whether CEM can serve as a more proportionate staging tool in selected scenarios.

Ultimately, the next step for preoperative contrast-enhanced breast imaging is not greater sensitivity, but greater decisional discipline. The goal should be to ensure that additional imaging information improves the proportionality of care rather than simply increasing the intensity of treatment.

## 10. Conclusions

Preoperative contrast-enhanced breast imaging has a substantial influence on surgical management in breast cancer. Its clinical value, however, cannot be defined by sensitivity alone. MRI and CEM do not simply detect more disease; they reshape the decisional environment in which surgery is planned.

A decision-oriented reading of the literature suggests that the impact of these techniques is best understood through three recurring patterns: refinement of surgical extent through improved disease mapping, pre-emptive escalation to avoid reoperation, and decision amplification through the detection of additional or indeterminate disease. These patterns help explain why the same imaging modality may support more proportionate surgery in some settings, such as invasive lobular carcinoma or assessment of nipple–areola complex involvement, while contributing to greater treatment intensity in others.

The central clinical issue is therefore not only whether contrast-enhanced imaging reveals more information, but whether that information is translated into better and more proportionate decisions. In unselected populations, routine use often appears to increase surgical intensity more clearly than it improves long-term oncologic outcomes. By contrast, selective, question-driven use appears most valuable when imaging addresses a clearly defined surgical uncertainty and when escalation is guided by confirmatory and multidisciplinary thresholds.

Preoperative contrast-enhanced imaging should thus be regarded as a decision-shaping tool, not merely a more sensitive diagnostic test. Its greatest contribution will come not from seeing more, but from helping clinicians act more proportionately on what is seen.

### Key Clinical Messages

- Preoperative contrast-enhanced breast imaging should be understood as a decision-shaping tool, because its principal effect is to alter surgical planning rather than simply improve lesion detection.
- Greater imaging sensitivity does not inherently translate into better clinical outcomes; the value of additional findings depends on how, and under what conditions, they modify treatment decisions.
- Invasive lobular carcinoma is the setting in which preoperative MRI appears to provide the most consistent decision-level benefit, particularly by improving disease mapping and reducing repeat surgery without systematically increasing mastectomy.
- In ductal carcinoma in situ, reduced re-excision rates must be interpreted cautiously because they are often accompanied by higher initial mastectomy rates.
- Additional lesions, especially MRI-only findings and non-mass enhancement, should not automatically trigger surgical escalation; histologic confirmation should precede irreversible management change whenever feasible.
- Assessment of nipple–areola complex involvement is one of the clearest high-value indications for contrast-enhanced imaging, as it may safely support nipple-sparing approaches in selected patients.
- Contrast-enhanced mammography may offer a different balance between information gain and downstream burden, but it does not eliminate the need for explicit thresholds linking detection to action.
- A value-based approach requires radiologists, surgeons, and multidisciplinary teams to focus not only on what imaging reveals, but on whether acting on those findings is likely to improve meaningful patient outcomes.

## Figures and Tables

**Table 1:** Recurrent Decision Patterns Associated with Preoperative Contrast-Enhanced Breast Imaging.

Decision Pattern	Typical Clinical Context	Main Imaging Contribution	Immediate Management Consequence	Potential Clinical Benefit	Main Clinical Risk
Refinement through improved disease mapping	Most evident in invasive lobular carcinoma; selected feasibility questions such as nipple–areola complex assessment	More accurate delineation of true disease extent or exclusion of occult involvement of critical structures	Better alignment of initial surgery with pathologic extent; selective modification of surgical approach	Reduced repeat surgery; improved selection for breast-conserving or nipple-sparing surgery; more proportionate operative planning	Overestimation of extent if imaging findings are not interpreted within a defined clinical context
Pre-emptive escalation to avoid reoperation	Particularly ductal carcinoma in situ; also mixed invasive populations in which margin concerns strongly influence planning	Broader depiction of lesion extent before surgery	More extensive initial surgery, often with lower tolerance for uncertainty or close margins	Fewer re-excisions or fewer secondary procedures in selected patients	Higher initial mastectomy rates; apparent improvement in reoperation outcomes achieved through upfront escalation rather than improved conservation
Decision amplification through additional or indeterminate disease	Unselected early breast cancer; MRI-only additional lesions; non-mass enhancement; contralateral findings	Detection of additional ipsilateral, contralateral, or indeterminate enhancing lesions	Additional imaging, second-look ultrasound, biopsy, multidisciplinary reassessment, and possible expansion of surgery	Identification of otherwise occult malignancy; possible avoidance of undertreatment in selected cases	Diagnostic cascades, anxiety, and escalation of surgery without consistent evidence of long-term oncologic benefit

**Table 2:** Clinical Contexts in Which Preoperative Contrast-Enhanced Imaging Is More or Less Likely to Add Decision-Level Value.

Clinical Context	Likely Decision-Level Value	Why Imaging May Be Useful	Main Caution	Suggested Clinical Posture
Invasive lobular carcinoma	High	Conventional imaging often underestimates extent; MRI more often reveals surgically relevant multifocal or multicentric disease	Additional findings should still be interpreted within a defined operative strategy	Selective but liberal use of MRI is reasonable when extent uncertainty may affect feasibility of breast conservation or operative planning
Ductal carcinoma in situ	Selective/cautious	MRI may improve delineation of extent and reduce radiologic underestimation	Reduced re-excision may be offset by increased first-line mastectomy; biologic significance of broader enhancement remains uncertain	Use selectively, especially when conventional imaging is discordant or extent is unclear; avoid automatic escalation based on imaging extent alone
Assessment of nipple–areola complex involvement	High for focused feasibility questions	MRI may help exclude NAC involvement and support nipple-sparing surgery in selected cases	Imaging should complement, not replace, clinicopathologic judgment	Question-driven use is strongly supported when the imaging result directly informs NSM eligibility
Unselected early invasive breast cancer	Limited for routine use	MRI may detect additional disease and alter surgical planning	Higher rates of more extensive surgery without consistent improvement in long-term oncologic outcomes	Avoid routine use as a universal staging tool; reserve imaging for clearly defined clinical uncertainties
MRI-only additional lesions or non-mass enhancement	Variable and often uncertain	May reveal occult disease not visible on conventional imaging	May trigger biopsies, delays, and escalation in cases with uncertain decision value	Require confirmatory pathway whenever feasible before irreversible surgical change
Contrast-enhanced mammography in preoperative planning	Moderate/context-dependent	May provide functional staging information with fewer indeterminate findings and less downstream workup than MRI	Evidence base is smaller; does not eliminate the problem of how findings should change management	Consider as an alternative functional imaging tool in selected settings, especially when access, workflow, or burden of workup are relevant

**Table 3:** What Preoperative Contrast-Enhanced Imaging Can and Cannot Reliably Do in Surgical Decision-Making.

Clinical Domain	What Imaging Can Reliably Do	What Imaging Cannot Reliably Do
Disease extent assessment	Improve delineation of lesion extent and identify multifocal, multicentric, or contralateral disease	Determine by itself whether greater extent necessarily requires more extensive surgery
Invasive lobular carcinoma	Reduce underestimation of disease extent and better align surgery with pathologic distribution	Guarantee improved long-term oncologic outcomes in all patients
Ductal carcinoma in situ	Improve anatomic delineation and reduce radiologic underestimation	Distinguish by itself which lesions truly require escalation rather than conservative management
Nipple–areola complex assessment	Support surgical feasibility assessment, including selected nipple-sparing approaches	Replace clinicopathologic judgment as a stand-alone criterion for nipple preservation
Additional lesions/MRI-only findings	Identify imaging abnormalities that may warrant further workup or biopsy	Independently justify irreversible surgical escalation without confirmatory integration
CEM/MRI enhancement patterns	Provide functional information that may flag increased uncertainty or potential underestimation	Predict biological aggressiveness or long-term progression with sufficient certainty to determine management alone

**Table 4:** Key studies informing the decision-oriented interpretation of preoperative contrast-enhanced breast imaging in breast cancer.

Study	Design	N	Clinical Setting	Imaging Modality	Key Quantitative Finding	Main Management Implication	Main Limitation
Marinovich et al. [1]	Multicentre prospective observational study	387 women with newly diagnosed early breast cancer	Selected patients in whom MDT practice considered MRI useful for treatment planning	Preoperative MRI	Treatment plan changed in 51% (95% CI, 46–56%); breast surgery plan changed in 31% (95% CI, 26–36%); planned mastectomy increased from 15% to 28% (RD 13%, 95% CI, 9–17); changes considered pathology-justified in 85% of evaluable cases	MRI substantially altered surgical planning in selected patients, often with increased mastectomy, but most changes were considered appropriate	Observational design; selected population; no non-MRI comparator
Mattar et al. [6]	Systematic review and meta-analysis of RCTs	5 randomized trials	Newly diagnosed invasive breast cancer	Preoperative MRI	Across RCTs, MRI did not consistently improve surgical outcomes; COMICE: reoperation 19% vs. 19%; MONET: re-excision 34% vs. 12%; POMB: reoperation 5% vs. 15%; BREAST-MRI: mastectomy increased by 8%, with no local relapse-free survival or overall survival benefit	Routine MRI was not supported as a means to improve overall surgical outcomes and may increase mastectomy risk	Heterogeneity of trial populations, endpoints, and MRI indications
Wang et al. [10]	Population-based observational cohort (SEER-Medicare)	24,379 women aged 67–94 years with stage I/II breast cancer treated with BCS	Early-stage breast cancer	Preoperative MRI	Subsequent mastectomy: 3.2 vs. 4.1 per 1000 person-years, AHR 0.92 (95% CI, 0.70–1.19); breast cancer mortality: 5.3 vs. 8.7 per 1000 person-years, AHR 0.89 (95% CI, 0.73–1.08); no significant benefit overall	MRI was not associated with significantly improved overall mastectomy or mortality outcomes in the overall cohort	Observational design; older Medicare population; residual confounding possible
Lobbes et al. [13]	Retrospective population-based cohort	36,050 Dutch patients with cT1-4N0-3M0 breast cancer treated with primary surgery	Histology-specific surgical outcomes	Preoperative MRI	MRI used in 29.8%; in invasive ductal cancer, MRI associated with more primary mastectomy (OR 1.30, 95% CI, 1.22–1.39); in invasive lobular cancer, MRI associated with less primary mastectomy (OR 0.86, 95% CI, 0.76–0.99), fewer positive margins after BCS (OR 0.59, 95% CI, 0.44–0.79), and fewer secondary mastectomies (OR 0.61, 95% CI, 0.42–0.88); contralateral breast cancer detection increased (OR 3.55, 95% CI, 3.01–4.17)	MRI effect appears histology-dependent, with more favorable surgical impact in invasive lobular carcinoma than in ductal cancers	Retrospective design; indication bias; MRI referral not randomized
Ha et al. [14]	Retrospective propensity score–matched study	603 patients with invasive lobular carcinoma; 369 underwent MRI; 196 matched pairs	Invasive lobular carcinoma	Preoperative MRI	Additional lesions detected in 39.3%; 65.5% of these were malignant; management changed in 25.5%, with 89.4% of changes considered appropriate; repeat surgery reduced (OR 0.140, *p* < 0.001); no increase in initial mastectomy (OR 0.876, *p* = 0.528) or final mastectomy (OR 0.744, *p* = 0.151)	In ILC, MRI may reduce repeat surgery without increasing mastectomy rates	Retrospective design despite matching; single-disease subgroup
Melvin et al. [16]	Observational cohort	110 patients with newly diagnosed invasive lobular carcinoma	Staging MRI in ILC	Preoperative MRI	Clinically significant additional lesions in 45%; targeted ultrasound ± biopsy in about one-third; ipsilateral additional lesions malignant in 58% vs. contralateral lesions in 8%; MRI changed surgery from BCS to mastectomy in 10%, all confirmed appropriate on final histopathology; BCS reoperation rate 11%; additional work-up associated with a 9-day relative delay to surgery	MRI identified substantial additional disease burden in ILC and changed surgery in a clinically relevant minority, but with some added work-up burden and delay	No non-MRI comparator; retrospective design; selection bias
Balleyguier et al. (IRCIS) [20]	Randomized controlled trial	352 analyzable women with biopsy-proven DCIS	Local DCIS planned for breast-conserving surgery	Preoperative MRI	Re-intervention within 6 months: 20% in MRI arm vs. 27% in control arm; absolute difference 7% (95% CI, −2% to 16%); per-protocol difference 9%, OR 0.59 (95% CI, 0.35–1.0; *p* = 0.05); total mastectomy 18% vs. 17%	MRI did not show sufficient surgical improvement to support routine use in this DCIS setting	Trial effect not clearly clinically decisive; limited to selected DCIS presentations
Cozzi et al./MIPA study [21]	Matched observational study	1005 women with pure unilateral DCIS; 309 matched pairs	Pure DCIS diagnosed at CNB/VAB	Preoperative MRI	First-line mastectomy 20.1% vs. 11.0% (OR 2.03, *p* = 0.003); reoperation 10.0% vs. 22.0% (OR for reoperation 0.40; OR for avoiding reoperation 2.53); overall mastectomy 23.3% vs. 17.8% (*p* = 0.111)	In matched DCIS patients, MRI lowered reoperation but increased first-line mastectomy	Observational design; residual confounding remains possible despite matching
Fancellu et al. [23]	Meta-analysis	9 studies; 1077 women with MRI and 2175 without MRI	DCIS	Preoperative MRI	Initial mastectomy increased (OR 1.72, *p* = 0.012; adjusted OR 1.76, *p* = 0.010); no significant difference in positive margins after BCS (OR 0.80, *p* = 0.059), reoperation for positive margins (OR 1.06, *p* = 0.759), or overall mastectomy rate (OR 1.23, *p* = 0.340)	MRI in DCIS was not associated with improved overall surgical outcomes	Heterogeneous studies; study-level adjustment only
Ozcan et al. [12]	Retrospective single-institution observational study	593 women with stage I–III breast cancer; 322 MRI vs. 271 no-MRI	Early-stage breast cancer with survival follow-up	Preoperative MRI	Bilateral cancers 5.3% vs. 3.7%; total mastectomy 57.8% vs. 51.3% (*p* = 0.12); margin positivity 6.2% vs. 7.4% (*p* = 0.63); recurrence 10.2% vs. 7.0% (*p* = 0.20); death 8.1% vs. 7.7% (*p* = 0.88); 5-year RFS HR 1.05 (95% CI, 0.67–1.67), adjusted HR 0.87 (95% CI, 0.53–1.43); 5-year OS HR 0.94 (95% CI, 0.51–1.74), adjusted HR 0.78 (95% CI, 0.40–1.52)	MRI was not associated with improved margin status, recurrence-free survival, or overall survival	Baseline imbalance between MRI and no-MRI groups; retrospective design
Taylor et al. [28]	Prospective comparative study	59 women with invasive breast cancer	Head-to-head preoperative staging comparison	CEM vs. MRI	Index lesion detection: MRI 97% vs. CEM 99%; 41 additional lesions in 29 patients; among 6 malignant additional lesions, MRI detected 6/6 and CEM 1/6; PPV for additional lesions: MRI 23% vs. CEM 8%; MRI yielded almost twice as many false positives as CEM	MRI may detect more otherwise occult additional malignant foci than CEM, particularly multifocal/multicentric disease, but with higher false-positive burden	Small sample; direct surgical impact not formally assessed; verification bias unavoidable
Montrognon et al. [30]	Retrospective cohort	132 patients with biopsy-confirmed invasive early breast cancer	Preoperative staging of early breast cancer	CEM	First surgical plan modified in 25%; surgery changed only because of CEM in 18.5%; surgery canceled in favor of neoadjuvant chemotherapy in 6%; primary tumor procedure enlarged in 12.1%; lymph node management changed in 17.4%; no increase in surgical delay (median 0 days)	CEM contributed meaningfully to surgical planning without prolonging time to surgery	Retrospective design; no MRI comparator in the same cohort

## Data Availability

No new data were created or analysed in this study. Data sharing is not applicable to this article.

## Abbreviations

The following abbreviations are used in this manuscript:

CEM	Contrast-enhanced mammography
DCIS	Ductal carcinoma in situ
DFS	Disease-free survival
ILC	Invasive lobular carcinoma
MRI	Magnetic resonance imaging
NAC	Nipple–areola complex
NME	Non-mass enhancement
OS	Overall survival
